# Characterizing SERCA Function in Murine Skeletal Muscles after 35–37 Days of Spaceflight

**DOI:** 10.3390/ijms222111764

**Published:** 2021-10-29

**Authors:** Jessica L. Braun, Mia S. Geromella, Sophie I. Hamstra, Holt N. Messner, Val A. Fajardo

**Affiliations:** 1 Department of Kinesiology, Brock University, St. Catharines, ON L2S 3A1, Canada; jb15gq@brocku.ca (J.L.B.); mg14dd@brocku.ca (M.S.G.); sh14iu@brocku.ca (S.I.H.); hm16og@brocku.ca (H.N.M.); 2 Centre for Bone and Muscle Health, Brock University, St. Catharines, ON L2S 3A1, Canada; 3 Centre for Neuroscience, Brock University, St. Catharines, ON L2S 3A1, Canada

**Keywords:** spaceflight, calcium handling, phospholamban, sarcolipin, neuronatin, muscle fiber type, sarcoplasmic reticulum, calcium ATPase, unloading

## Abstract

It is well established that microgravity exposure causes significant muscle weakness and atrophy via muscle unloading. On Earth, muscle unloading leads to a disproportionate loss in muscle force and size with the loss in muscle force occurring at a faster rate. Although the exact mechanisms are unknown, a role for Ca^2+^ dysregulation has been suggested. The sarco(endo)plasmic reticulum Ca^2+^ ATPase (SERCA) pump actively brings cytosolic Ca^2+^ into the SR, eliciting muscle relaxation and maintaining low intracellular Ca^2+^ ([Ca^2+^]_i_). SERCA dysfunction contributes to elevations in [Ca^2+^]_i_, leading to cellular damage, and may contribute to the muscle weakness and atrophy observed with spaceflight. Here, we investigated SERCA function, SERCA regulatory protein content, and reactive oxygen/nitrogen species (RONS) protein adduction in murine skeletal muscle after 35–37 days of spaceflight. In male and female soleus muscles, spaceflight led to drastic impairments in Ca^2+^ uptake despite significant increases in SERCA1a protein content. We attribute this impairment to an increase in RONS production and elevated total protein tyrosine (T) nitration and cysteine (S) nitrosylation. Contrarily, in the tibialis anterior (TA), we observed an enhancement in Ca^2+^ uptake, which we attribute to a shift towards a faster muscle fiber type (i.e., increased myosin heavy chain IIb and SERCA1a) without elevated total protein T-nitration and S-nitrosylation. Thus, spaceflight affects SERCA function differently between the soleus and TA.

## 1. Introduction

It is well established that microgravity exposure during spaceflight comes with a great deal of physiological and psychosocial challenges that can compromise astronaut health [1,2,3]. Loss of muscle mass and strength is an important factor that can impede the astronaut’s ability to perform mission-related duties during space travel and upon return to Earth or partial gravity (i.e., Moon or Mars). Mammals (i.e., humans and rodents) have evolved with the never-ending downward pull of gravity on Earth, and therefore postural muscles such as the soleus are known to be most affected with spaceflight. Similar to unloading models on Earth, spaceflight and microgravity exposure in rodents unloads the postural soleus causing extensive muscle atrophy and a fiber-type shift from slow-oxidative to fast-glycolytic [4,5,6,7,8,9]. Similar changes have also been observed in human soleus muscles after 17 days of spaceflight [10]. Not surprisingly, the reduction in muscle mass has also been associated with muscle weakness [10]. However, a recent study highlights the fact that skeletal muscle unloading causes disproportionate losses in muscle mass and strength, with the decline in muscle strength occurring at a faster rate than muscle mass [11]. While this suggests that the muscle weakness caused by unloading is not merely due to a reduction in muscle size, the underlying cellular mechanisms behind this disproportionate loss in muscle force are still poorly understood, and some have suggested a role for Ca^2+^ dysregulation and increased reactive oxygen/nitrogen species (RONS) production [11,12].

The sarco(endo)plasmic reticulum Ca^2+^ ATPase (SERCA) pump is responsible for lowering cytosolic Ca^2+^ by actively bringing it into the sarco(endo)plasmic reticulum (SR) [13,14]. There are two main SERCA isoforms in skeletal muscle: SERCA1a and SERCA2a (the fast and slow isoforms, respectively) [15]. Partly consistent with the slow-to-fast fiber type transition, recent proteomic analyses have shown that ≈30 days of spaceflight significantly increased SERCA1a *mRNA* in the murine soleus, without altering SERCA2a *mRNA* [9]. This is similar to findings with rodent hindlimb unloading via tail suspension, the best accepted spaceflight analog, where an increase in SERCA1a protein accompanied by no changes in SERCA2a can be found in the unloaded soleus [16,17]. However, to our knowledge, the consequences of spaceflight specifically on SERCA function in murine skeletal muscle remains unknown. This is important as SERCA’s action not only works to elicit muscle relaxation but also to maintain low intracellular Ca^2+^ ([Ca^2+^]_i_). Impaired Ca^2+^ uptake by SERCA results in increased [Ca^2+^]_i_, which can lead to cytotoxic effects such as protein degradation, cell death, and elevated RONS production, subsequently causing muscle weakness and wasting [18,19,20,21].

In addition to SERCA isoform content, there are several other factors that can regulate SERCA function. SERCA has two main regulatory proteins in skeletal muscle, phospholamban (PLN) [15,22] and sarcolipin (SLN) [15,22]; however, we have also recently identified another regulator in muscle named neuronatin (NNAT) [23,24]. Each of these regulators work to reduce SERCA-mediated Ca^2+^ uptake either through reductions in Ca^2+^ affinity or causing Ca^2+^ ‘slippage’ back into the myoplasm [22,25]. In addition to these regulators, SERCA is also highly susceptible to RONS-mediated post-translational modifications, such as tyrosine (T)-nitration and cysteine (S)-nitrosylation [26,27,28], that can damage the pump and impair Ca^2+^ uptake [29,30]. Whether the expression of these proteins or the levels of T-nitration and S-nitrosylation in response to spaceflight are altered in muscle remains unknown. Thus, the purpose of our study was to characterize SERCA function and measure these regulatory proteins and post-translational modifications in murine skeletal muscle after 35–37 days of spaceflight. Both slow-twitch soleus and fast-twitch tibialis anterior (TA) muscles were analyzed to determine whether SERCA would be affected differently across the muscle types.

## 2. Results

### 2.1. SERCA Function in Soleus Muscles from Male and Female Spaceflown Mice

**Figure 1:**
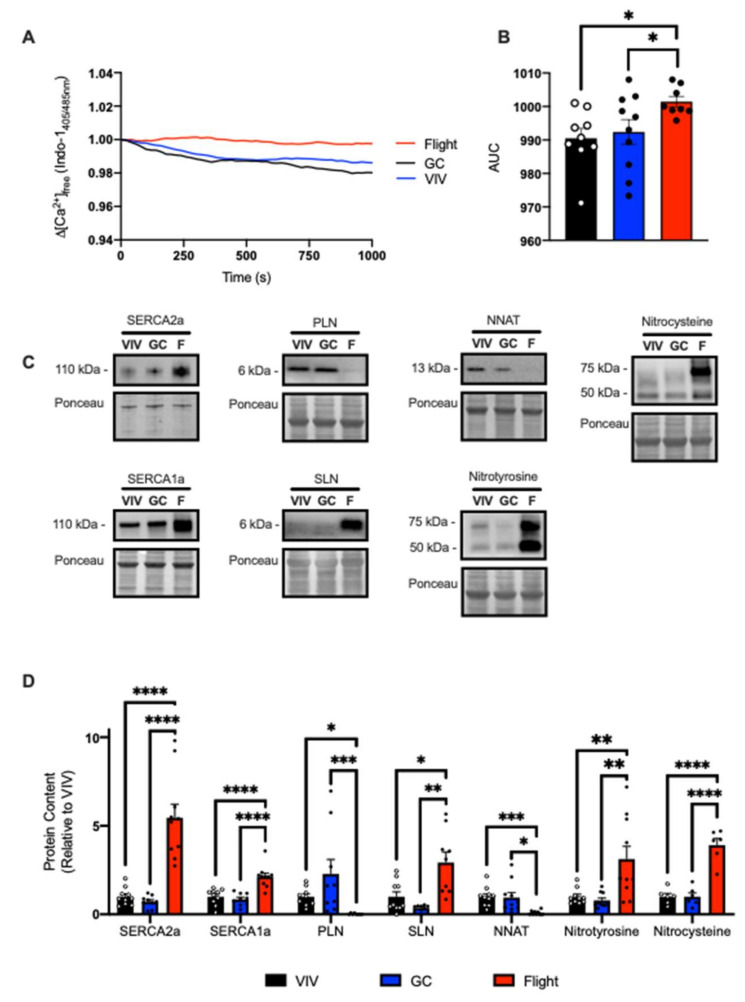
Spaceflight reduced Ca^2+^ uptake in the soleus from male mice. (**A**) Change in the ratio of Ca^2+^-bound to Ca^2+^-free Indo-1 (405/485 nm emission). (**B**) Area under the curve (AUC) measurements show significant increases in the flight group, representing impaired Ca^2+^ uptake. Representative Western blot images (**C**) and densitometric analysis (**D**) of SERCA2a/1a, PLN, SLN, NNAT, and total protein T-nitration and S-nitrosylation. All values are means ± SEM, and Western blot data are presented relative to VIV control. * *p* < 0.05; ** *p* < 0.01; *** *p* < 0.005; **** *p* < 0.0001 using a one-way ANOVA and Tukey’s post hoc test (*n* = 9–10 per group).

We first characterized SERCA function in murine soleus muscles obtained from NASA’s Rodent Research 9 (RR9) mission. These mice were aboard the International Space Station (ISS) for 33 days and exposed to microgravity for 35 days. Absolute soleus weights were smaller in the flight group compared with Vivarium (VIV) and Ground Controls (GC) (Table 1). Assessment of SERCA function in the RR9 soleus demonstrates that spaceflight caused significant impairments in the amount of ATP-dependent Ca^2+^ uptake with significantly elevated area under the curve (AUC) when compared to VIV and GC (Figure 1A,B). To investigate whether the reductions in Ca^2+^ uptake were due to changes in SERCA protein content, we employed Western blotting. Interestingly, we found significant increases in both SERCA2a and SERCA1a protein content in the flight group compared to VIV and GC (Figure 1C,D). We also found significant reductions in PLN and NNAT, but significant increases in SLN in the flight group compared to GC and VIV (Figure 1C,D). Due to limitations in the amount of sample available, we could not directly measure the amount of S-nitrosylation and T-nitration on the SERCA isoforms specifically. However, measuring total protein levels revealed that the flight group had significant elevations in both total protein S-nitrosylation and T-nitration compared to GC and VIV (Figure 1C,D).

**Figure 2:**
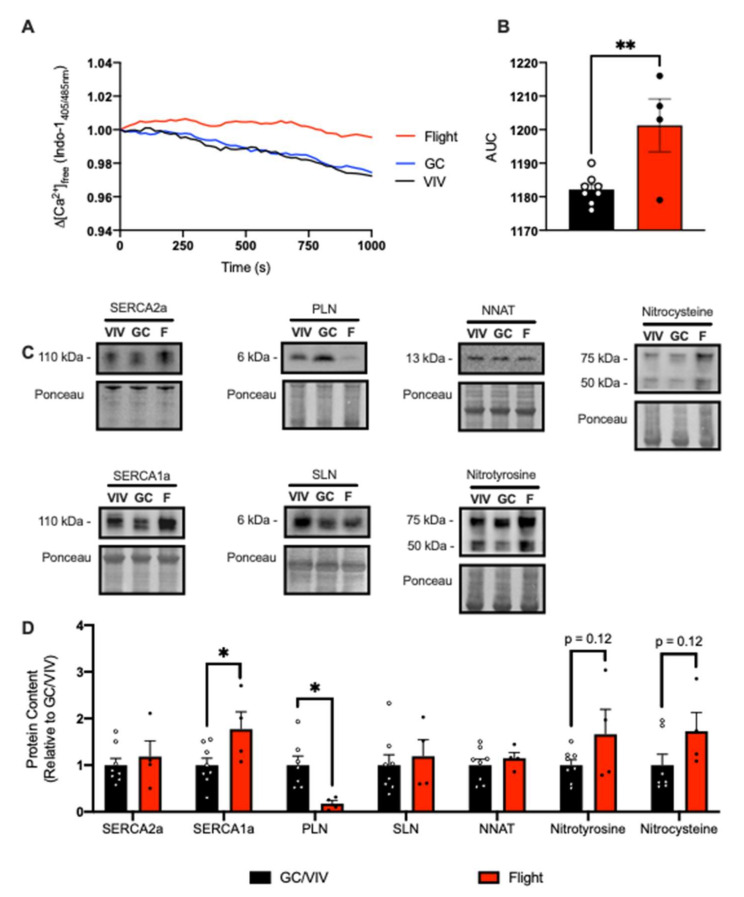
Spaceflight reduced Ca^2+^ uptake in the soleus from female mice. (**A**) Change in the ratio of Ca^2+^-bound to Ca^2+^-free Indo-1 (405/485 nm emission). (**B**) AUC was significantly increased in the flight group compared with GC/VIV representing reduced Ca^2+^ uptake. Representative Western blot images (**C**) and densitometric analysis (**D**) of SERCA2a/1a, PLN, SLN, NNAT, and total protein T-nitration and S-nitrosylation. All values are means ± SEM, and Western blot data are presented relative to GC/VIV control. * *p* < 0.05; ** *p* < 0.01 and values above bars indicate *p*-values using Student’s *t*-test (*n* = 4–8 per group).

In consideration of a potential effect of biological sex, we also obtained murine soleus samples from female mice from NASA’s Rodent Research 1 (RR1) mission. These mice were aboard the ISS for 33 days and exposed to microgravity for 37 days. Consistent with soleus muscles from male mice, soleus muscles from female mice were smaller in the flight group vs. GC and VIV (Table 1). However, since we were only able to obtain a limited number of samples (*n* = 4 per group), we combined GC and VIV groups for statistical comparisons as there were no differences revealed between these groups. Similar to the data obtained from male mice from the RR9 mission (Figure 1), we found that SERCA function was impaired with a significant reduction in Ca^2+^ uptake compared with GC/VIV (Figure 2A,B). While there were no differences observed in SERCA2a, we did find a significant increase in SERCA1a content as well as a significant decrease in PLN in the flight group vs. GC/VIV (Figure 2C,D). We did not find any changes in SLN or NNAT (Figure 2C,D). Similar to male mice from the RR9 mission, protein S-nitrosylation and T-nitration were higher in the flight group from the RR1 mission, albeit non-significantly (Figure 2C,D).

### 2.2. SERCA Function in TA Muscles from Male Spaceflown Mice

**Figure 3:**
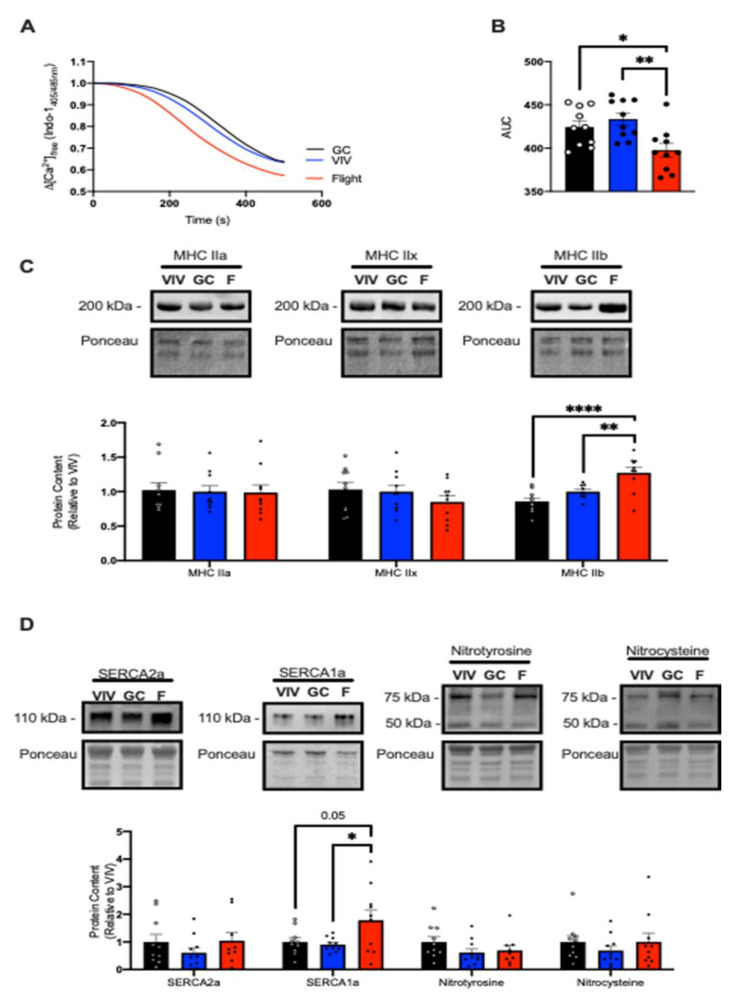
Spaceflight increased Ca^2+^ uptake and induced a shift towards a fast phenotype in the TA of male mice. (**A**) Change in the ratio of Ca^2+^-bound to Ca^2+^-free Indo-1 (405/485 nm emission). (**B**) AUC was significantly reduced in the flight group, representing increased Ca^2+^ uptake. (**C**) Representative Western blot images and densitometric analysis of myosin heavy chain (MHC) IIa, IIx, and IIb. (**D**) Representative Western blot images and analyses of SERCA isoform content and total protein T-nitration and S-nitrosylation. All values are means ± SEM, and Western blot data are presented relative to VIV control. * *p* < 0.05; ** *p* < 0.01; **** *p* < 0.0001; values above bars indicate *p*-values using a one-way ANOVA with Tukey’s post hoc test (*n* = 9–10 per group).

We next examined SERCA function in the fast-glycolytic TA muscle. Like the soleus muscle, TA muscles were smaller in the flight group compared with GC and VIV (Table 1). However, and in contrast with the slow-twitch soleus, SERCA assessment showed a significant increase in Ca^2+^ uptake in the flight group with a significant reduction in AUC compared to VIV and GC (Figure 3A,B). While fiber type transformations in the unloaded soleus have been well characterized, less is known regarding the potential changes in fiber type composition in the TA in response to spaceflight. Here, we found that the TA muscles from the flight group had no changes in myosin heavy chain (MHC) IIa or IIx content; however, there was a significant increase in MHC IIb compared with both VIV and GC (Figure 3C). When examining SERCA isoform content, we found no changes in SERCA2a, but there was a significant increase in SERCA1a content in the flight group vs. GC and VIV (Figure 3D). SERCA regulatory proteins, PLN, SLN, and NNAT, were not detected in the TA with up to a 40 µg of total protein loaded (data not shown). Furthermore, we did not find any changes in total protein S-nitrosylation and T-nitration (Figure 3D).

## 3. Discussion

To our knowledge, our study is the first to investigate SERCA function in soleus and TA muscles from mice exposed to microgravity via spaceflight. We observed that, in the postural soleus, Ca^2+^ uptake was significantly reduced in the flight group in both male and female mice. Associated with these findings were elevations in total protein T-nitration and S-nitrosylation, which could implicate elevated RONS production in the impairment of SERCA function in the postural soleus muscle during spaceflight. Contrarily, in the TA, we saw a significant enhancement in Ca^2+^ uptake in the flight group compared with GC and VIV, which was associated with a fast fiber type transition and no change in total protein T-nitration or S-nitrosylation. Thus, our findings reveal important differences on the effects of spaceflight on SERCA function between slow and fast muscle types.

Previous work with ground-based and spaceflight models in both rodents and humans have demonstrated that the postural soleus undergoes a fiber type shift towards a faster phenotype and that the muscle displays significant atrophy and weakness [3,5,7,10,12,17,31,32,33,34,35]. Furthermore, previous work has suggested a potential role for Ca^2+^ dysregulation [11,12] in the ensuing muscle atrophy and remodeling. Others have also shown changes in SERCA isoform mRNA levels with spaceflight and Earth-based unloading—suggesting that spaceflight can alter SERCA transcription [9,16,17]. Here, our study provides novel insight and is the first to demonstrate impaired SERCA Ca^2+^ uptake in the space-flown soleus of both male and female mice. Consistent with such work, we did observe a significant increase in SERCA1a protein content, the isoform predominantly associated with fast skeletal muscle [15]. It is important to note that irrespective of isoform, the primary determinant of Ca^2+^ uptake is SERCA density [36]. However, the increase in SERCA1a and SERCA2a could not overcome the stress of spaceflight in the postural soleus. While an increase in SERCA2a protein content was also observed, we speculate that this may be a compensatory response attempting to (but failing to) increase Ca^2+^ uptake in the face of impaired Ca^2+^ homeostasis.

Accompanying the changes in SERCA protein content, three SERCA regulators—PLN, SLN, and NNAT—were also investigated. PLN and NNAT are both highly conserved proteins primarily found in oxidative skeletal muscle [22,24,37]. PLN allosterically binds to SERCA and reduces its affinity for calcium [22,37], while NNAT has been shown to inhibit Ca^2+^ uptake and promote SERCA uncoupling in vitro [24]. In response to spaceflight, significant reductions in both PLN and NNAT protein content were seen, and thus we believe may not contribute to the impairments in SERCA function observed in the postural soleus. Further, since both PLN and NNAT are found primarily in oxidative skeletal muscle [22,24,37], the reduction in both of their protein contents is not entirely surprising given the fast fiber type shift. Conversely, we found that SLN was significantly upregulated in the soleus in response to spaceflight, which again was, to some extent, expected. SLN is a well-established SERCA uncoupler [25,38] that is upregulated in many muscle wasting conditions, including muscular dystrophy [39], sarcopenia [40], and soleus unloading [34,41]. SLN has been shown to promote calcineurin signaling via SERCA inhibition, thereby promoting the oxidative phenotype and muscle mass [42,43]. Its deletion has also been shown to cause more severe muscle atrophy and a more pronounced fast fiber type transition in the tenotomized soleus [34]. Thus, the increase in SLN presumably contributes to the impairment in SERCA function observed with spaceflight, but whether it contributes to muscle atrophy and weakness requires further investigation.

SERCA is highly susceptible to RONS-mediated post-translational modifications such as T-nitration and S-nitrosylation that can ultimately impair its ability to regulate cytosolic Ca^2+^ levels [26,27,28,29]. Despite not having enough sample to conduct SERCA-specific analysis of RONS modifications, our total protein assessment provides novel insight. Specifically, we found dramatic increases in total protein T-nitration and S-nitrosylation in the soleus muscles from the flight group vs. VIV and GC in the RR9 mission. We found similar effects in the soleus muscles from female mice in the RR1 mission, albeit non-significant presumably due to low sample size. Nevertheless, our results could suggest that elevations in RONS production contribute to the SERCA impairments observed in the soleus muscle after spaceflight. In fact, both Ca^2+^ and RONS act as signaling molecules inside the cell and their pathways interact; however, disruption in RONS can contribute to Ca^2+^ dysregulation and vice versa [44]. For example, it is known that chronically elevated [Ca^2+^]_i_ can increase RONS production by activating cytosolic NADPH oxidase enzymes and by causing mitochondrial dysfunction via increased mitochondrial Ca^2+^ uptake [45,46,47], ultimately perpetuating cellular dysfunction and pathology. On this note, a recent study has found that improving SERCA function through pharmacological activation reduces mitochondrial RONS production in both aged mice [48] and in a mouse model of elevated oxidative stress [19]. Taken together, our results may reveal a negative cyclical relationship where in response to spaceflight, elevated RONS production in the soleus may damage SERCA, contributing to less Ca^2+^ uptake, which only adds further to RONS production. This highlights the importance of determining whether improving SERCA function can alleviate soleus muscle weakness and atrophy observed during spaceflight.

We also attempted to examine a potential effect of biological sex, measuring SERCA function in soleus muscles from both male and female mice. Although we observed significant reductions in Ca^2+^ uptake in soleus muscles from space-flown male and female mice, there were some subtle differences in the changes in SERCA isoform, SERCA regulatory proteins, and total protein T-nitration and S-nitrosylation assessed via Western blotting. However, direct comparisons between male and female mice were prevented due to differences in sample size, sample preparation, and mission. Nonetheless, our study could point towards a potential effect of biological sex. Specifically, our results show that while SERCA2a and SERCA1a were upregulated in soleus muscles from male space-flown mice, only SERCA1a was upregulated in soleus muscles from female mice exposed to microgravity. With the importance of SERCA density [36], this blunted upregulation in SERCA2a could contribute to the impairments in SERCA function as well as the ensuing muscle atrophy. Similar to male mice, spaceflight in female mice significantly reduced PLN in the soleus, which is important since overexpression of PLN can lead to severe muscle atrophy [49], and thus we view this reduction in both male and female mice as an adaptive response. In contrast, NNAT and SLN protein levels were unaltered in female space-flown mice. Indeed, several studies have shown that SLN protein/mRNA is upregulated in atrophic/myopathic conditions [34,39,42,43,49,50,51], albeit most of these studies have been conducted in male mice, and thus our findings highlight the importance of including both male and female mice in future studies exploring the effects of spaceflight, unloading, disuse, disease, and aging on muscle.

In addition to characterizing SERCA function in the postural soleus, we also investigated the effects of spaceflight in the fast-glycolytic TA muscle. TA muscles were smaller in the flight group and had significantly elevated MHC IIb protein levels. Thus, similar to the soleus muscle, it appears that spaceflight causes a reduction in TA muscle size and a fast fiber type shift. However, unlike the soleus muscle, we found that SERCA function was enhanced in the TA muscle in response to spaceflight. We attribute this effect to the slow-to-fast fiber type shift that came with a significant increase in SERCA1a protein content, and perhaps more importantly, no change in total protein T-nitration or S-nitrosylation. That is, unlike the soleus muscle, TA muscles displayed no signs of elevated RONS production. In turn, the reduction in muscle size in the soleus was accompanied by an impairment in SERCA function and increased RONS production, whereas the reduction in TA muscle size was accompanied by an improvement in SERCA function and no alterations in RONS. The exact reasons explaining this difference between slow and fast muscles are unknown, but we speculate that it may be due to differences in duty. The postural soleus functions as a chronically active and loaded muscle, whereas the TA muscle is more phasic in its activation and load, being used for more explosive type movements. For this reason, the impact of muscle unloading would be more prominent in the postural soleus. It is also important to note that the reduction in TA muscle size may be simply due to the reduction in body mass observed in the RR9 flight group vs. the GC and VIV (Appendix B Figure A2) and not actually representative of atrophy per se. Though normalizing soleus to body mass removed any significant differences between groups, we also noticed a trending increase in TA/body mass ratio in the flight vs. VIV groups. However, these results are limited as a more accurate measure of muscle size would be myofiber cross-sectional area (CSA). In this respect, it has been recently shown that spaceflight significantly reduces muscle fiber cross-sectional area (CSA) in the murine soleus both in absolute and relative to body mass measures [7]. In contrast, the fast glycolytic extensor digitorum longus (EDL) muscle showed no reductions in absolute or relative CSA after 91 days of spaceflight. Therefore, we speculate that like the EDL, the TA muscle from 35 days of spaceflight would not display any reductions in CSA, suggesting that there is no actual atrophy occurring in this muscle type. Future studies should investigate this further in addition to examining TA muscle contractility to determine whether the improvement in SERCA function observed in this muscle would lead to improvements in force production.

## 4. Materials and Methods

### 4.1. Muscle Samples

**Figure A1:**
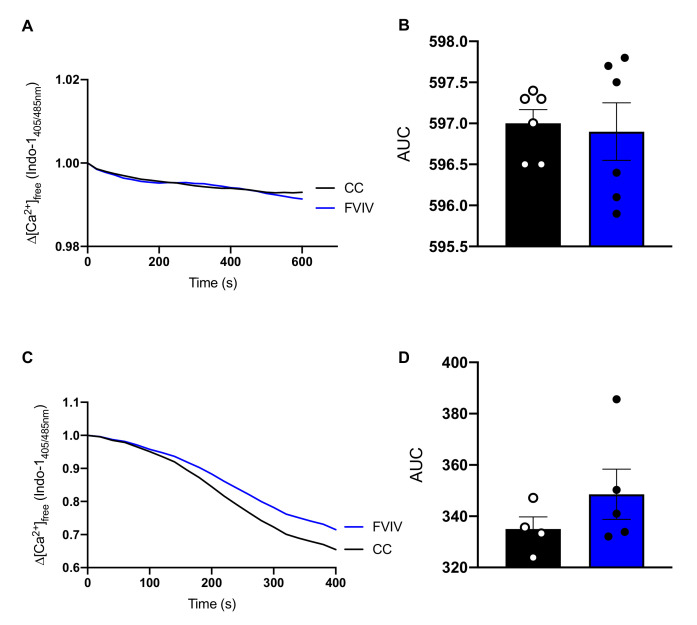
Ca^2+^ uptake assays in cohort control 1 and 2 (CC1 and CC2) from soleus (**A**,**B**) and TA (**C**,**D**) muscles. Area under the curve analyses revealed no differences between CC1 and CC2 for soleus or TA. All values are means ± SEM.

**Figure A2:**
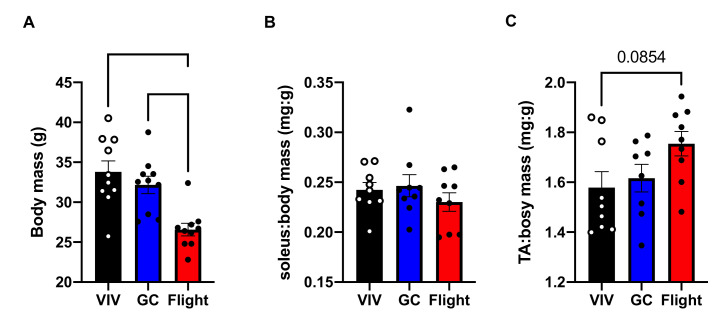
Body mass (**A**), soleus/body mass (**B**), and TA/body mass (**C**) data from flight, GC, and VIV groups from the RR9 mission. All values are means ± SEM. Values above bars indicate *p*-values using a one-way ANOVA and Tukey’s post hoc test (*n* = 10 per group).

Soleus and TA muscle samples were obtained from the NASA Life Sciences Data Archive Institutional Scientific Collection Biospecimen Sharing Program. We were specifically provided with soleus and TA samples from male C57BL/6J mice from the RR9 mission (*n* = 10 per group) and soleus samples from female C57BL/6J mice from the RR1 mission (*n* = 4 per group). The male mice from the RR9 mission were 10 weeks of age at launch, and the female mice from the RR1 mission were 16 weeks of age. All mice originated from Jackson Laboratories. Mice in the flight group and ground control group were housed in NASA’s Rodent Flight Hardware [52] and were provided ad libitum access to food (NASA Type 12 Nutrient upgraded rodent food bars [53]) and water. The rodent habitat was modified from heritage flight hardware to provide long-term housing for rodents aboard the ISS (see Supplementary Materials Figure S4 from [54]) and was programmed on a 12:12 h light/dark cycle with enough food and water being provided so that replenishment was not required for the mission. Temperature, humidity, and carbon dioxide levels were matched for flight and ground control groups [52,54]. Mice in the VIV group were housed in standard laboratory cages. For RR9, we received TA muscles that were snap frozen in liquid nitrogen and stored at −80 °C, and soleus muscles that were stored in RNALater (ThermoFisher Scientific, Waltham, MA, USA) at −80 °C. For RR1, we received soleus muscles that were snap frozen in liquid nitrogen and stored at −80 °C. For all samples, we homogenized the muscles in homogenizing buffer (250 mM Sucrose, 5 mM HEPES, 0.2 mM PMSF, 0.2% NaN_3_ (pH 7.5)) prior to storing them at −80 °C. In addition to the flight, GC, and VIV groups, we also received two cohort control groups from the RR9 mission. Due to Hurricane Irma (September 2017), the original RR9 GC and VIV experiments were prematurely terminated. The GC and VIV experiments were then repeated in May 2018 using the same strain of mice that were used for the flight experiment. Along with the new GC and VIV groups, an additional set of mice were used as cohort controls to normalize the variation due to differences in cohorts. That is, the mice originally dedicated to serve as the VIV group in 2017 were labeled as cohort 1, and another set of similarly matched (both in age, sex, and treatment) mice were run as cohort 2 in 2018. Importantly, we found no differences in Ca^2+^ uptake in either the soleus or TA muscles from cohort control 1 (CC1) and cohort control 2 (CC2) (Appendix A Figure A1), and therefore did not need to normalize to any variation due to cohorts.

### 4.2. Calcium Uptake

Rates of Ca^2+^ uptake in the muscle homogenates were measured using the Indo-1 Ca^2+^ fluorophore as previously described [38,49,55,56], but fitted onto a 96-well plate [24]. Briefly, muscle homogenate was added to reaction buffer (200 mM KCl, 20 mM HEPES, 10 mM NaN_3_, 5 µM TPEN, 15 mM MgCl_2_ (pH 7.0)) containing Indo-1 (4 µM final concentration; 57180, Sigma-Aldrich, St. Louis, MO, USA). Samples were then plated in duplicate, to which ATP (10 mM final concentration) was added to initiate Ca^2+^ uptake. The ratio of Ca^2+^-bound to Ca^2+^-free Indo-1 (405/485 nm emission) was measured using a Molecular Devices M2 plate reader (Molecular Devices, San Jose, CA, USA) upon excitation at 355 nm at 37 °C. The amount of Ca^2+^ uptake was then calculated as the change in the ratio of Ca^2+^-bound to Ca^2+^-free Indo-1 and measuring the area under the curve, with the smaller the area under the curve being indicative of more Ca^2+^ uptake over time.

### 4.3. Western Blotting

To assess SERCA2a, SERCA1a, PLN, SLN, and NNAT protein content as well as the amount of post-translational modifications—nitrotyrosine and nitrocysteine—present in the soleus, we used Western blotting [23,24,29]. Using a BCA assay to assess protein concentration, we utilized a total protein load of 2.5 mg, 10 mg, 20 mg, 25 mg, and 15 mg for the aforementioned proteins, respectively, and 20 mg for nitrotyrosine and nitrocysteine in the soleus muscle. For the TA, 10 mg of protein was loaded for SERCA2a and 2.5 mg for SERCA1a with 20 mg similarly loaded for post-translational modifications. To investigate the MHC protein content, we loaded 8 mg of protein. Muscle homogenates were solubilized in Laemmli buffer (#161-0747, BioRad, Hercules, CA, USA) before being separated by SDS-PAGE using TGX BioRad PreCast 4–15% gradient gels (#4568086, BioRad) and transferred to a polyvinylidene difluoride (PVDF) membrane using the BioRad Transblot Turbo for all except for SLN, which was separated using tricine-based SDS-PAGE and transferred to a nitrocellulose membrane using a wet-transfer. All membranes were blocked using Everyblot (#12010020, BioRad) for 5 min at room temperature before the addition of primary antibodies. SERCA1a (Ma3-911), SERCA2a (MA3-919), and PLN (MA3-922) antibodies were obtained from ThermoFisher Scientific (Waltham, MA, USA). SLN antibodies were purchased from Sigma Aldrich (ABT13) and NNAT from Proteintech (26905-1-AP, Rosemont, IL, USA). Nitrotyrosine was purchased from Cayman Chemical (189542, Ann Arbor, MI, USA), and nitrocysteine was obtained from Abcam (ab94930, Cambridge, UK). Finally, the antibodies for MHC IIa (SC-71), MHC IIx (6H1), and MHC IIb (10F5) were purchased from Developmental Studies Hybridoma Bank (Iowa City, IA, USA). All primary antibodies were incubated overnight at 4 °C. Membranes were washed 3× in Tris-buffered saline + 0.1% (*v/v*) Tween 20 (TBST) prior to 1 h incubation at room temperature with the corresponding anti-mouse (SERCA2a, SERCA1a, PLN, nitrotyrosine, nitrocysteine) or anti-rabbit (SLN, NNAT) secondary antibodies diluted in 5% (*w/v*) milk in TBST. Following another 3 washes in TBST, membranes were imaged using Immobilon^®^ ECL Ultra Western HRP Substrate (MilliporeSigma, Burlington, MA, USA) and a BioRad ChemiDoc Imager. Ponceau stains were used to quantify total protein loads, and all images were analyzed using ImageLab software (BioRad).

### 4.4. Statistics

All data are presented as means ± standard error of the mean (SEM). A one-way ANOVA with Tukey’s post hoc test was used to compare the VIV, GC, and flight groups with respect to Ca^2+^ uptake via area under the curve analyses as well as protein contents. Student’s *t*-test was used to compare flight vs. combined GC and VIV for the RR1 soleus as well as for comparisons between the two cohort control groups. Statistical significance was set at *p* ≤ 0.05, and outliers were detected using the ROUT method (Q = 2%) and were removed prior to analyses. All statistical tests were employed using GraphPad Prism 8.

## 5. Conclusions

We investigated SERCA function in the soleus and TA muscles of space-flown mice. We saw reductions in Ca^2+^ uptake and increases in RONS in the soleus. In contrast, we found a significant enhancement in Ca^2+^ uptake, a fast fiber type shift with increased MHC IIb and SERCA1a, and no changes in RONS in the TA. Future studies should further examine the role of biological sex on SERCA function and whether protecting SERCA function can resist the atrophy and weakness observed in the soleus muscles with spaceflight.

## Figures and Tables

**Table 1:** Absolute muscle mass (mg) from NASA Rodent Research 1 (RR1) and Rodent Research 9 (RR9) missions.

	VIV	GC	Flight
RR9 *Tibialis anterior*	53.4 ± 5.0 *	53.0 ± 3.4 *	47.3 ± 5.7
RR9 *Soleus*	8.2 ± 1.2 ***	7.9 ± 0.9 **	6.1 ± 0.7
RR1 *Soleus*	7.0 ± 0.5 **	7.0 ± 0.4 **	5.0 ± 0.8 ^1^

^1^ All values are means ± standard error of the mean (SEM). * *p* < 0.05; ** *p* < 0.01; *** *p* < 0.005 using a one-way ANOVA with Tukey’s post hoc test (*n* = 4 per group, RR1; *n* = 9–10 per group, RR9). VIV, vivarium control; GC, ground control.

## Data Availability

The data is available on NASA’s Life Sciences Data Archive, found at https://lsda.jsc.nasa.gov (released in 2022).
